# Benzalkonium Chloride Poisoning in Pediatric Patients: Report of Case with a Severe Clinical Course and Literature Review

**DOI:** 10.3390/toxics12020139

**Published:** 2024-02-08

**Authors:** Viorela Nițescu, Andreea Lescaie, Dora Boghițoiu, Coriolan Ulmeanu

**Affiliations:** 1 “Carol Davila” University of Medicine and Pharmacy, 020021 Bucharest, Romania; andreea.lescaie@drd.umfcd.ro (A.L.); dora.boghitoiu@umfcd.ro (D.B.); coriolan.ulmeanu@umfcd.ro (C.U.); 2 “Grigore Alexandrescu” Clinical Emergency Hospital for Children, 017443 Bucharest, Romania

**Keywords:** disinfectants, quaternary ammonium compounds, benzalkonium compounds, poisoning, child, aspiration, pneumonia, pneumothorax, corrosion

## Abstract

The use of disinfectants, particularly those containing quaternary ammonium compounds (QUACs), has dramatically escalated globally since the coronavirus disease 2019 pandemic. We report a case that highlights the risks associated with ingesting low-concentration QUAC solutions and emphasize the importance of effective management in resolving severe lesions without sequelae. A 17-month-old boy experienced severe respiratory failure after ingesting a disinfectant containing benzalkonium chloride (BAC). The child was initially treated at a local emergency department and was subsequently transferred to a pediatric poison center. Upon evaluation, the child was found to have grade III-A corrosive esophageal lesions and chemical pneumonitis. Several complications, including massive pneumothorax and candidemia, occurred during the clinical course of the disease. However, with timely medical intervention and appropriate supportive care, the patient completely recovered without any long-term sequelae. The properties of BAC and the comprehensive management approach may have been responsible for the patient’s full recovery, despite the potentially life-threatening effects of ingesting disinfectants.

## 1. Introduction

The global spread of severe acute respiratory syndrome coronavirus 2 (SARS-CoV-2) has led to a substantial increase in the use of disinfectants [1,2,3].

The United States Environmental Protection Agency has published a list of disinfectants proven to be effective against SARS-CoV-2 [4]. Approximately half of these products contain quaternary ammonium compounds (QUACs) as active ingredients [5,6]. Although QUACs have been used as disinfectants and sanitizers since 1930 [6], their use by the general population has significantly increased due to public awareness regarding their efficacy against SARS-CoV-2 [2,5].

As a result of the increased use of disinfectants, there has been a corresponding rise in the number of cases of poisoning with these substances. According to poison control centers, there was a significant increase in the number of reports during the pandemic, with rates of 16.4% in the United States, 60.4% in South Africa, and 121.5% in France [7,8,9,10,11]. In addition, the severity of disinfectant poisoning worsened during the pandemic, with a greater number of symptomatic cases having been observed [1,9]. 

Unfortunately, the chemical structure of these disinfectants and their intended purposes have not been examined in the majority of studies. Only du Plessis et al. [8] and Yasseen et al. [11] make exceptions, with the former reporting that hand sanitizers were the primary cause of disinfectant poisoning and the latter categorizing exposures to hand sanitizers and other disinfectants. Therefore, the pandemic’s influence on the number of patients poisoned with QUACs cannot be accurately assessed. 

Benzalkonium chloride (BAC), a cationic surfactant with well-documented antimicrobial properties [4,6], is a QUAC. BAC can displace divalent cations from the lipid bilayers of bacterial cell membranes, including those of human cells [6,12,13].

It was initially believed that the toxicological profile of BAC was limited to local effects due to its high elimination via feces, which was assumed to be related to its absorption rate from the gastrointestinal tract being less than 10% [4]. However, Seguin et al. [14] characterized the metabolic pathways of BAC with respect to hepatic CYP enzymes, providing an alternative explanation for its high elimination in the stool. The presence of BAC in blood samples serves as evidence of systemic exposure [2,15].

The manifestations of BAC largely overlap with those of alkali compounds, with corrosive tissue damage being the predominant characteristic [16,17]. However, BAC toxicological profiles have revealed specific effects, including apoptosis-induced lung injury [12,16], bronchoconstriction [18], and hypersensitivity reactions [19,20,21,22].

The clinical and biological features of BAC poisoning are derived from sporadic case reports and case series, as observational studies regarding QUAC exposure are lacking. Herein, we report a case to contribute to the body of knowledge regarding the hazards of disinfectant ingestion in children, focusing on the risks associated with the use of QUACs.

## 2. Case Description

A 17-month-old boy ingested an unknown quantity of disinfectant while riding in a car seat. The disinfectant was stored in a sippy-cap water bottle within the child’s reach and was intended for the disinfection of objects. Upon ingestion, the mother promptly induced vomiting and rushed the child to the nearest emergency department. Fortunately, the mother retained the original bottle, enabling the identification of the disinfectant’s chemical composition and properties (Table 1).

The patient’s condition rapidly deteriorated at the local emergency department, resulting in severe respiratory failure due to glottic edema. The patient required intubation and mechanical ventilation to ensure adequate respiration. Following clinical stabilization, our poison center was contacted, and the child was subsequently transferred to our clinic via air ambulance.

The patient was admitted to our intensive care unit five hours after ingestion. On physical examination, the patient was comatose, intubated, and mechanically ventilated. The patient was febrile (38.4 °C), with a heart rate of 150 beats per minute, normal blood pressure of 94/54 mmHg, and normal urinary output. The chest was clear on auscultation, and no adventitious sounds were heard. A cardiac examination revealed normal heart sounds. The abdominal wall palpatory examination results were normal, including the limits of the liver and spleen. However, an oral cavity examination revealed intense oropharyngeal edema, hyperemia, and massive swelling of the uvula. No pathological dermatologic changes were observed.

**Figure 1:**
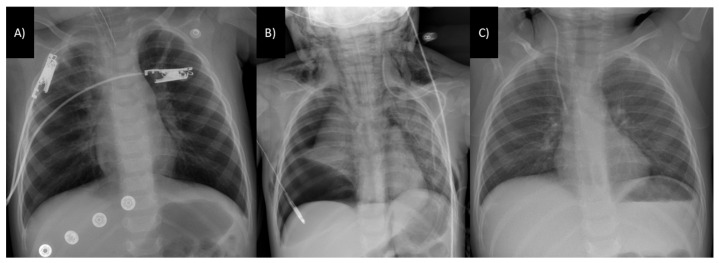
Chest radiographs: (**A**) upon admission, a chest radiograph depicted bilateral perihilar alveolar infiltration; (**B**) chest radiograph performed on hospital day seven showed subcutaneous emphysema, pneumomediastinum, and massive right pneumothorax; (**C**) at discharge, the chest radiograph displayed a normal aspect.

The patient’s initial blood test results indicated metabolic acidosis (pH 7.25; base excess: −7.7 mmol/L), an increased white blood cell count (17,190 cells/mm^3^—reference range: 4000–12,000 cells/mm^3^) with a predominance of neutrophils (84%), and an elevated creatine kinase concentration (238 U/L—reference range: 0–171 U/L). Bacterial cultures obtained at admission were negative, except for positive results for coagulase-negative *Staphylococcus* on auricular and cutaneous swabs and *Streptococcus pneumoniae* on nasal swabs. The initial chest radiograph revealed bilateral perihilar alveolar infiltration (Figure 1A).

**Figure 2:**
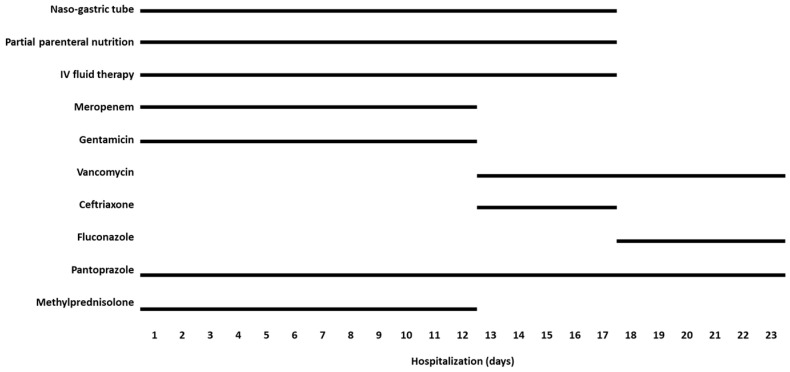
Treatment during hospitalization. The patient’s treatment was complex and included partial parenteral nutrition, broad-spectrum antibiotics, and corticosteroids. A specific treatment was added for *Candida* bloodstream invasion. Abbreviations: IV, intravenous.

The severity of the lesions was assumed to be significant given the hazardous nature of the substance [24,25,26] and the presence of clinical and laboratory risk factors [27,28,29,30]. Therefore, treatment was initiated immediately (Figure 2). A nasogastric tube was inserted, and partial parenteral nutrition was started, including amino acids, glucose, and lipid emulsion. According to established guidelines [31,32], the patient was administered 2 mg/kg/day of pantoprazole intravenously (IV), 10 mg/kg/day of methylprednisolone IV, and a broad-spectrum antibiotic regimen consisting of meropenem at 50 mg/kg/day IV and gentamicin at 4 mg/kg/day IV.

The use of corticosteroids has been debated in several studies [31,32,33]. Currently, the European Society of Gastrointestinal Endoscopy and the European Society for Pediatric Gastroenterology Hepatology and Nutrition guidelines endorse their use for grade IIB lesions and suggest their possible implementation for grade III lesions [34].

**Figure 3:**
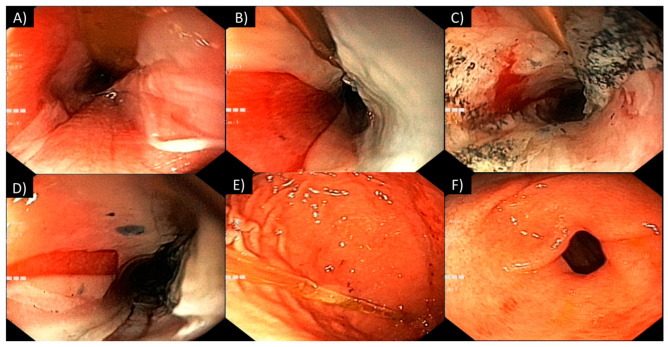
Superior digestive endoscopy performed 16 h after ingestion: (**A**–**D**) the esophagus exhibited patchy necrosis, ulceration, and sloughing; (**E**,**F**) the gastric mucosa displayed prominent hyperemia and bleeding marks.

Based on state-of-the-art practices [31,32], an endoscopic procedure was performed 16 h after ingestion, revealing the presence of grade III-A lesions according to the Zargar classification [35]. The patient’s mucosal edema diminished, exposing extensive areas of sloughing lesions in the oral cavity; pharynx; and vocal cords and bleeding spots on the tonsils. The esophagus exhibited patchy necrosis, ulceration, and sloughing (Figure 3A–D). The gastric mucosa displayed prominent hyperemia and bleeding marks (Figure 3E,F).

**Figure 4:**
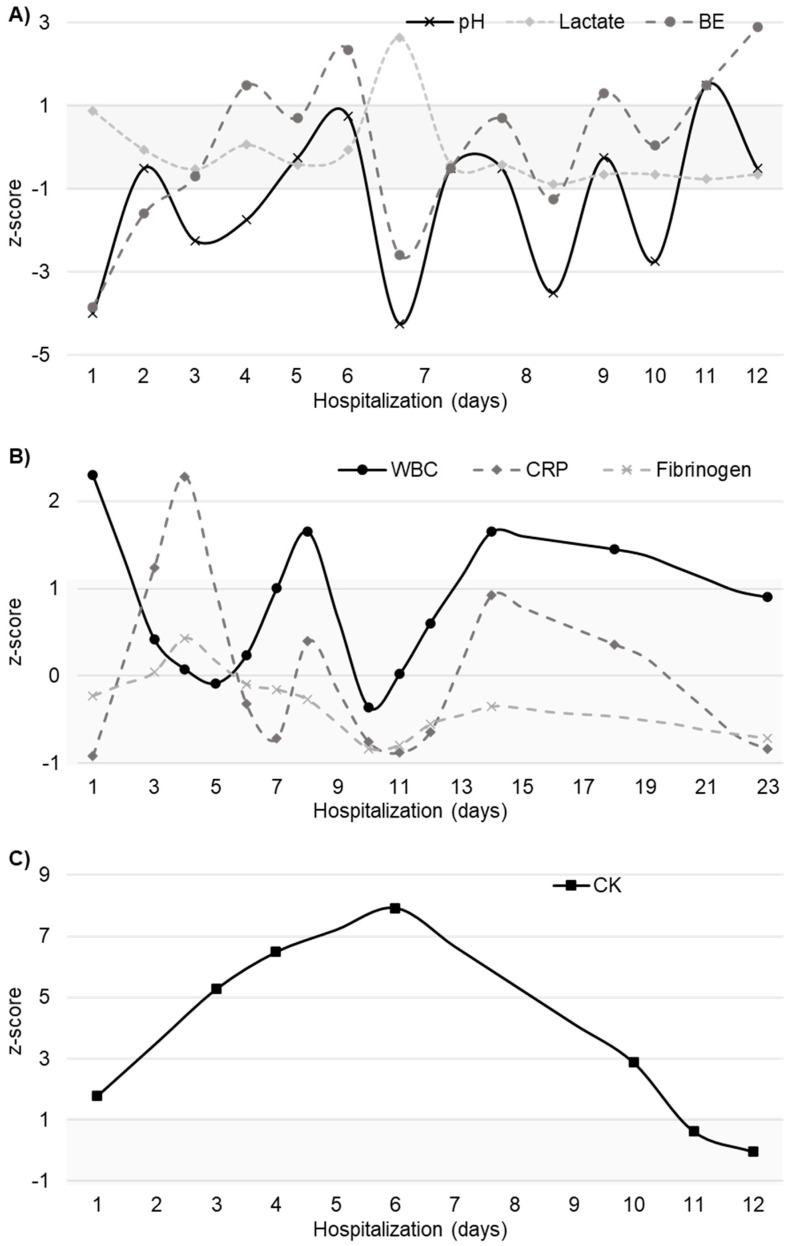
Laboratory test results during the clinical course: (**A**) venous blood gas (VBG) analysis; (**B**) leukocyte count and inflammatory markers; and (**C**) creatine kinase level. Note: The VBG and creatine kinase levels are illustrated for the initial 12 days, up until the point of normalization. Abbreviations: BE, base excess; WBC, white blood cell count; CRP, C-reactive protein; CK, creatine kinase.

Biological samples were collected periodically to monitor patient progress and modify treatment plans as necessary. The laboratory test results are presented kinetically in Figure 4. Notable increases in inflammatory markers were detected four days after ingestion.

The patient’s lung damage progressively worsened, ultimately resulting in the development of subcutaneous emphysema, pneumomediastinum, and massive right pneumothorax on hospital day seven (Figure 2B), necessitating urgent drainage-tube implantation. Subsequently, the patient’s lung damage gradually improved, as confirmed through blood tests and chest radiography on hospital day 10. The patient was then successfully extubated. Thoracic angio-computed tomography revealed improvements in the patient’s lung status, with only a small condensation in the right upper lobe. On hospital day 12, the patient was transferred to the toxicology department. 

During the initial 24 h in the toxicology department, the patient’s status deteriorated, with fever and sleepiness. The central venous catheter (CVC) was removed, and microbiological CVC cultures (negative) and blood cultures (positive for *Candida parapsilosis*) were performed. Based on these findings, 40 mg/kg/day of IV vancomycin was administered for 10 days, followed with 5 days each of 100 mg/kg/day of IV ceftriaxone and 7 mg/kg/day of IV fluconazole (Figure 3). 

The patient’s response to treatment was favorable. On hospital day 18, the nasogastric tube was successfully removed, and the patient was able to resume oral alimentation, which was initiated with clear liquids and progressed to double-mixed alimentation. 

**Figure 5:**
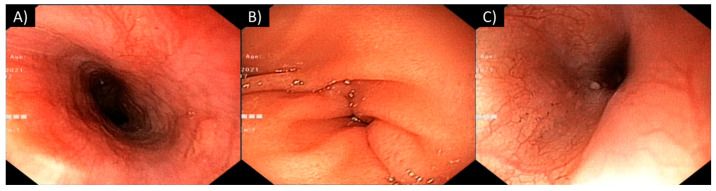
Superior digestive endoscopy performed 21 days after ingestion showed resolution of corrosive lesions with persistence of (**A**) punctiform necrosis in the esophagus and (**B**,**C**) hyperemic gastric mucosa.

A follow-up endoscopy conducted on hospital day 21 revealed a significant improvement in the patient’s condition, with small ulcerations on the tongue, an edematous pharynx, ulcerations (<0.5 cm), punctiform necrosis in the esophagus, and hyperemic mucosa in the stomach (Figure 5).

On hospital day 25, the patient was discharged in stable condition. The patient had a normal appetite, normal swallowing function, and normal laboratory values, except for iron-deficiency anemia. At home, the child received semi-liquid nourishment, oral iron therapy, and vitamin B supplementation.

Three months after ingestion, physical examination, blood test, and chest radiography results were within the normal ranges. A barium meal examination revealed normal swallowing function without any signs of gastroesophageal reflux. Endoscopy revealed a complete recovery without complications.

## 3. Discussion

### 3.1. General Considerations

The exposure characteristics of our patient were similar to those in most reported cases of unintentional disinfectant poisoning [24,36]. The primary contributing factors to disinfectant poisoning in this patient were the inappropriate storage of the hazardous chemical and its accessibility to the child [37,38]. This report contributes to several studies that have highlighted the absence of parental awareness regarding the hazards of corrosive substances, coupled with the absence of preventive measures, as a significant issue [39,40,41]. The tasteless and odorless properties of alkaline liquid solutions, which can lead to the ingestion of a significant volume before protective reflexes intervene, were additional contributing factors in this case [27]. 

The role of poison control centers is essential to monitoring exposure to substances and issuing warnings to the general public, authorities, and manufacturers. These warnings can result in increased public awareness and regulatory market measures, ultimately serving as an effective preventive measure.

### 3.2. Gastrointestinal Tract Involvement

The patient presented in this report had severe corrosive lesions in the esophagus, necessitating prompt multidisciplinary management to ensure favorable outcomes [18,40,42]. The resolution of the mucosal lesions was consistent with the expected timeframe for BAC poisoning [15]. Despite the serious nature of the esophageal lesions, the current patient had a favorable outcome that did not include the development of strictures, which is noteworthy given the abundance of risk factors typically observed in patients with alkali poisoning [27,31,42].

A literature search regarding the prevalence of strictures in patients with BAC poisoning was conducted to identify factors that may have contributed to a favorable outcome. One observational study reported the rates of stricture development in patients with BAC poisoning [17]. This study included 11 children who had ingested BAC among 968 cases of corrosive poisoning, and none of the patients developed strictures [17]. However, the previous study did not report the burn severity.

In a review of cases of both adults [16,43,44] and children [45,46,47,48,49] who survived the ingestion of BAC solutions, strictures were described in only a few neonatal cases [48]. Therefore, the positive outcome in our patient and the management or nature of the substance involved are likely not directly nor causally related. 

### 3.3. Respiratory Tract Involvement

The rapid development of acute respiratory distress in this patient may be related to laryngeal edema and BAC-induced bronchoconstriction through the release of mediators from mast cells and the stimulation of cholinergic receptors [16]. The mechanisms of lung toxicity have been extensively studied via animal models [18], cell culture investigations [12], and forensic reports [16], resulting in the characterization of BAC-induced lung injury. Based on these data and previously reported cases [43,46,47,48,49], we concluded that our patient developed chemical pneumonitis because he aspirated the BAC solution.

The worsening of our patient’s condition that occurred on hospital day seven had not been reported in cases of QUAC-related poisoning. This may be due to the occurrence of a delayed toxic event facilitated by apoptosis, which is the primary mechanism underlying BAC-induced lung injury [18]. However, it is more likely that the incident was related to prolonged mechanical ventilation, as demonstrated by Amigoni et al. [50], who reported that chemical injury in one lung may lead to mechanical ventilation-induced injury in the contralateral lung.

### 3.4. Other Complications

The patient in this case report experienced candidemia as an indirect consequence of BAC poisoning. According to Chirica et al. [51], bloodstream microbial invasion and elevated inflammatory markers are indicative of tardive esophageal rupture. However, this was not observed in the current patient. Candidemia may have resulted from prolonged hospitalization in the intensive care unit.

### 3.5. Previously Reported Cases of BAC Ingestion

At the time of the literature review, 22 pediatric cases of ingested BAC had been published. Of these, 6 were neonatal cases and were featured in a descriptive study conducted by Turan et al. [48], another 11 cases were included in a descriptive study by Karaman et al. [17], and the remaining cases were documented in case reports (Table 2). Conversely, only three adult cases involving oral exposure to BAC have been documented (Table 2). In all instances, the concentration of the BAC solution was found to be at least 10% [16,44,45,47,49], except for a single adult case where a solution with a concentration of less than 10% was ingested [43]. Currently, this case report is the only available report that suggests that even low concentrations of BAC can lead to severe symptoms.

Chemical lung injury was observed in more than half of the patients. Concurrently, esophageal corrosive lesions were reported in almost all patients, but the majority did not experience long-term consequences [16,44,45,47,49]. All pediatric cases had a favorable outcome, whereas both of the adults who had intentionally ingested the substance were reported as deceased, which highlights the greater severity of intentional poisoning compared with unintentional poisoning [16,44,45,47,49].

### 3.6. Pharmacological Properties

Several studies have described the environmental toxicity of QUACs and their impacts on humans exposed to them for extended periods [12,52,53,54,55]. However, the pharmacological endpoints associated with acute ingestion or pulmonary aspiration have been less studied.

Research with animal models has demonstrated that orally administering BAC results in high bioavailability and rapid distribution to various tissues, with dose-dependent profiles [14,56]. The maximum concentration of BAC can be observed in most tissues 24 h post-administration, with immediate distribution to the lungs [14,56]. Additionally, studies by Mohapatra et al. [57] have shown that QUACs exhibit significant bioaccumulation, suggesting the potential for prolonged systemic exposure following ingestion.

The median lethal dose for ingestion exceeds 200 mg/kg but can decrease to as low as 8.5 mg/kg in cases of pulmonary aspiration [18,58].

This pharmacological property suggests that the potential of BAC solutions to cause lung injury, both through systemic exposure and direct corrosion, should not be overlooked.

### 3.7. Proposed Management of BAC Poisoning

This case report, along with previous ones, suggests that patients who have ingested BAC exhibit symptoms similar to those experienced by patients who have been exposed to caustic substances. However, BAC ingestion can lead to allergic glottis edema, which may lead to acute respiratory failure. This condition is not frequently observed in individuals who have consumed corrosive substances and may cause medical personnel to divert their attention from evaluating corrosive lesions.

The first step in medically managing BAC poisoning is represented by a comprehensive assessment of the affected systems, with a particular focus on the respiratory symptoms. Advanced life support measures should be applied as needed, including mechanical ventilation.

A chest radiograph should be obtained at the earliest possible time to evaluate the extent of the chemical lung injury, and it should be repeated at least every 12–24 h in the first days, or more frequently if clinically indicated. Moreover, given the potential for lung injuries resulting from systemic exposure, patients who have ingested BAC require close and prolonged respiratory monitoring to promptly diagnose and treat any complications.

To ensure that corrosive injuries are not overlooked, it is essential to perform an esogastroscopy within the first 24–48 h. Additionally, the esogastroscopy must be performed again 21–28 days post-ingestion to assess the healing process of the lesions and the occurrence of sequelae.

The treatment plan should be based on the extent and severity of the lesions. It may involve intensive monitoring and intervention to address fluid, electrolyte, and acid–base imbalances; parenteral nutritional support; broad-spectrum antibiotics; or corticosteroids. 

A positive outcome can be achieved by comprehensively assessing and diagnosing all complications upon admission and throughout the hospital stay, as well as promptly treating all complications, including concurrent infections.

However, given the scarcity and diversity of available data in the literature, more research and reports are needed to validate an effective management strategy for BAC poisoning.

## 4. Conclusions

The ingestion of disinfectants is a significant public health concern, which can result in severe and potentially life-threatening effects. The presented patient developed acute respiratory failure, chemical pneumonitis, and severe caustic esophagitis. Despite a severe clinical course complicated by massive pneumothorax, the patient ultimately recovered without any long-term impairment. The management of BAC poisoning needs to be comprehensive, with a focus on preserving vital functions and addressing injuries. The favorable outcomes of the patient may be attributable to the properties of BAC or the comprehensive management approach. Clinical toxicologists must be aware of the potential, yet rare, hazards posed by low-concentration QUAC solutions and exercise caution in managing such cases.

## Figures and Tables

**Table 1:** The composition and chemical–physical properties of the disinfectant at 20 °C [23].

**Active ingredients:**	15.6% Cocospropylene diamine–guanidine diacetate
	35% Phenoxypropanols
	2.5% Benzalkonium chloride
**pH:**	9.1–9.5
**Density:**	0.99 g/cm^3^
**Viscosity:**	30 mPa*s

Abbreviations: °C, degree Celsius; g, gram; cm^3^, cubic centimeter; mPa, millipascal; s, second.

**Table 2:** Circumstances and clinical outcomes of BAC-poisoned patients reported in the literature.

	Okan [49]	Civan [47]	Wilson [45]	Wilson [45]	Bekdas [46]	Kumar [43]	Tambuzzi [16]	Spiller [44]
Age	2 days	2 days	2 months	2 months	2 months	42 years	61 years	78 years
Sex	F	F	M	F	M	M	F	M
Intentional	No	No	No	No	No	No	Yes	Yes
Concentration (%)	10	10	11	11	10	<10	10	10
Esophageal corrosive injury	Yes	Yes	No	No	Yes	Yes	Yes	Yes
Lung injury	Yes	No	Yes	No	Yes	Yes	Yes	Yes
Received corticoids	Yes	No	No	No	Yes	Yes	Yes	Unknown
Received antibiotics	Yes	Yes	Yes	No	Yes	Yes	No	Unknown
Outcome	Favorable	Favorable	Favorable	Favorable	Favorable	Favorable	Deceased	Deceased
Sequels	No	Unknown	No	No	No	No	NA	NA

Abbreviations: F, female; M, male; BAC, benzalkonium chloride; NA, not applicable.

## Data Availability

All data are available in the article body.
